# Supplementary material for: Development and validation of a simple clinical nomogram for predicting infectious diseases in pediatric kidney transplantation recipients: a retrospective study
Source: PeerJ. 2024 Nov 21;12:e18454. doi: 10.7717/peerj.18454 (PMC11586046; doi:10.7717/peerj.18454)
Supplement: Table S1 [file peerj-12-18454-s001.docx]

**Table S1. Immunosuppressant Use in Infected and Non-Infected Pediatric Kidney Transplant Recipients**

| **Medicine** | **All patients**  **N = 297** | **Infected**  **N=164** | **Non-infected N=133** |
| --- | --- | --- | --- |
| **CNI+MM+ Corticosteroids** | 174 | 87 | 87 |
| **Tacrolimus + Mycophenolate mofetil + Methylprednisolone** | 9 | 8 | 1 |
| **Tacrolimus + Mycophenolate mofetil + Prednisone** | 69 | 31 | 38 |
| **Tacrolimus + Enteric-coated mycophenolate sodium+ Methylprednisolone** | 8 | 4 | 4 |
| **Tacrolimus + Enteric-coated mycophenolate sodium + Prednisone** | 79 | 39 | 40 |
| **Cyclosporine A + Mycophenolate mofetil + Prednisone** | 8 | 5 | 3 |
| **Cyclosporine A + Enteric-coated mycophenolate sodium + Prednisone** | 1 | 0 | 1 |
| **CNI+MM** | 117 | 72 | 45 |
| **Tacrolimus + Mycophenolate mofetil** | 68 | 43 | 25 |
| **Tacrolimus + Enteric-coated mycophenolate sodium** | 44 | 24 | 20 |
| **Cyclosporine A + Mycophenolate mofetil** | 4 | 4 | 0 |
| **Cyclosporine A + Enteric-coated mycophenolate sodium** | 1 | 1 | 0 |
| **CNI+ Mizoribine** | 5 | 4 | 1 |
| **Tacrolimus + Mizoribine** | 5 | 4 | 1 |
| **CNI+ Mizoribine+ Corticosteroids** | 1 | 1 | 0 |
| **Tacrolimus + Mizoribine + Prednisone** | 1 | 1 | 0 |

CNI: Calcineurin Inhibitors; Mycophenolate Mofetil (MM);
