# Supplementary figures and images for: Development and validation of a simple clinical nomogram for predicting infectious diseases in pediatric kidney transplantation recipients: a retrospective study
Source: PeerJ. 2024 Nov 21;12:e18454. doi: 10.7717/peerj.18454 (PMC11586046; doi:10.7717/peerj.18454)

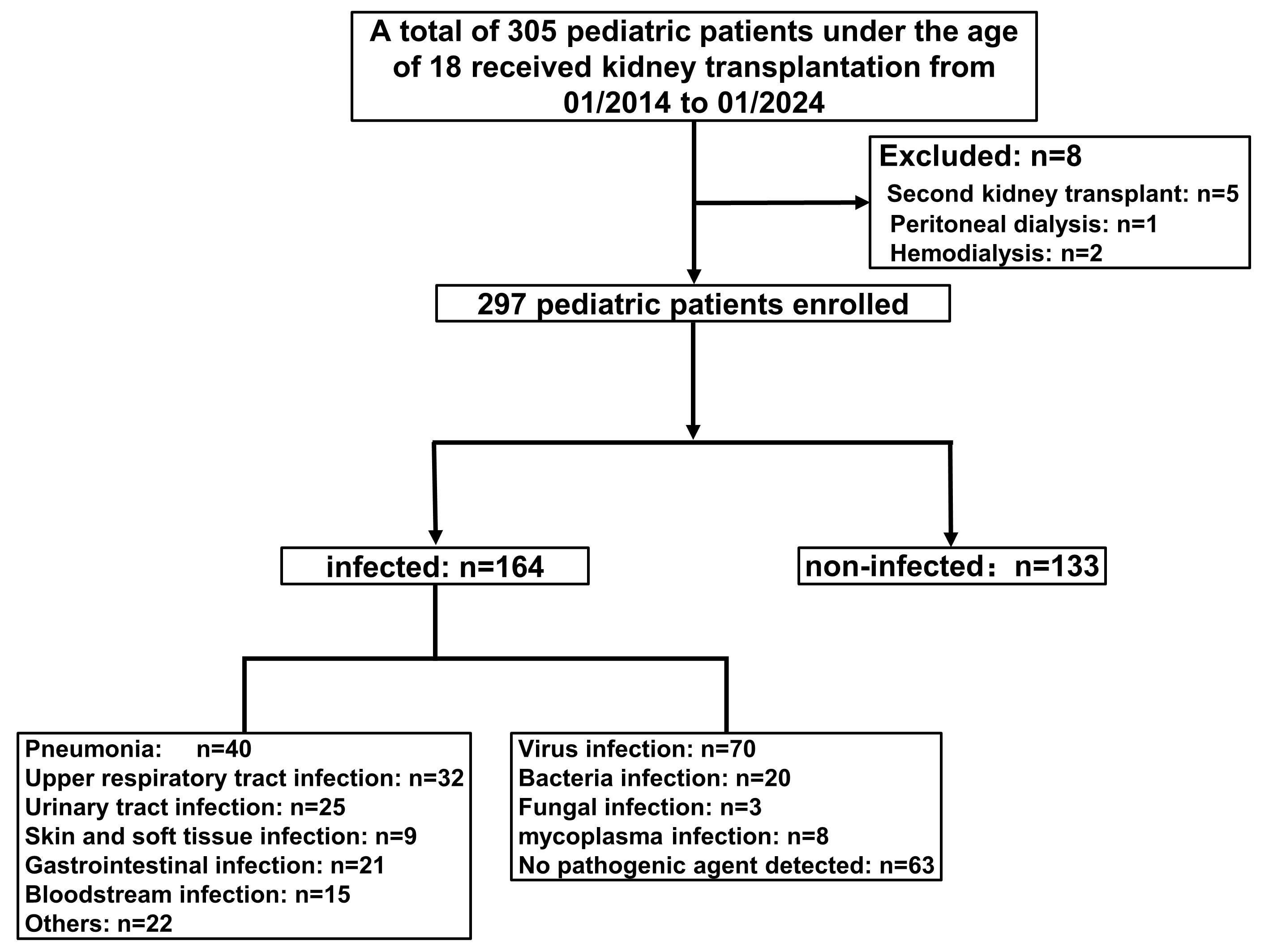

Supplement: Supplemental Information 2 [file peerj-12-18454-s002.zip › Figures and data/Figure1/Fig1-new.tif]

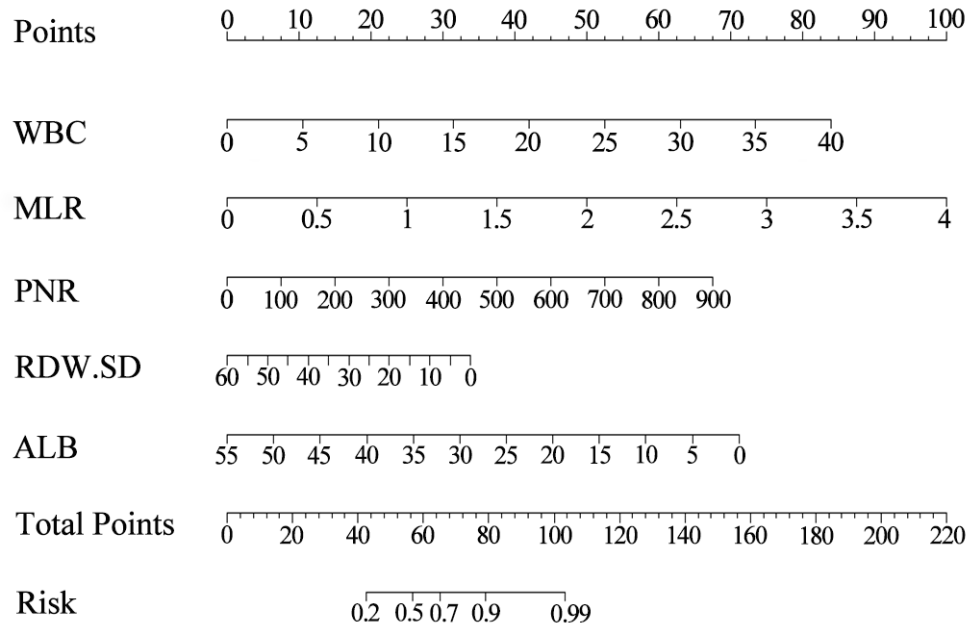

Supplement: Supplemental Information 2 [file peerj-12-18454-s002.zip › Figures and data/Figure2/Fgures2.pdf]

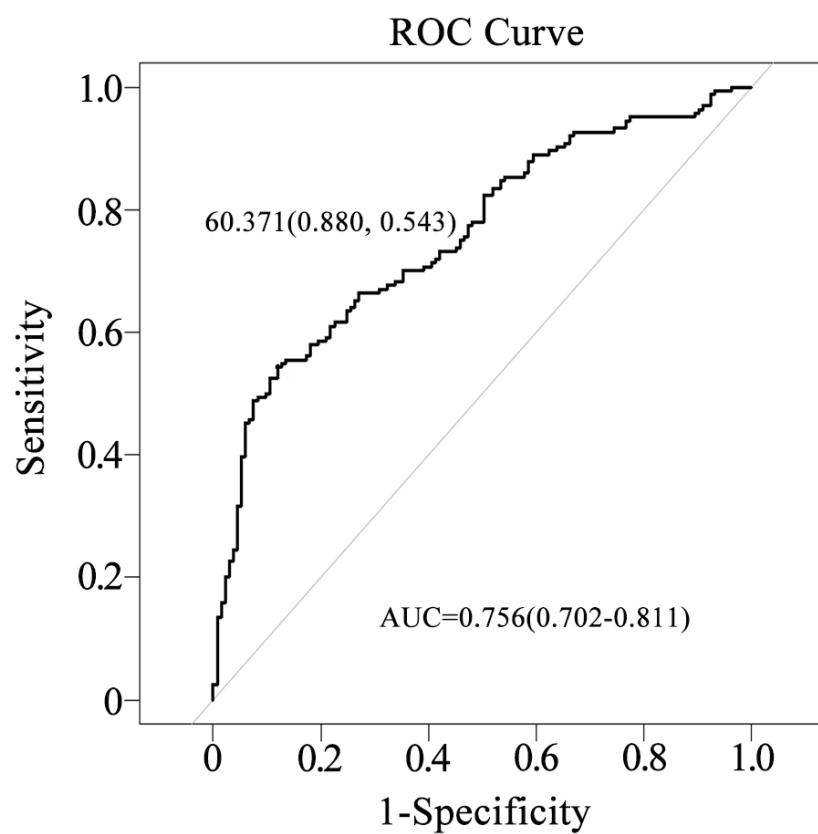

Supplement: Supplemental Information 2 [file peerj-12-18454-s002.zip › Figures and data/Figure3/Fgures3.pdf]

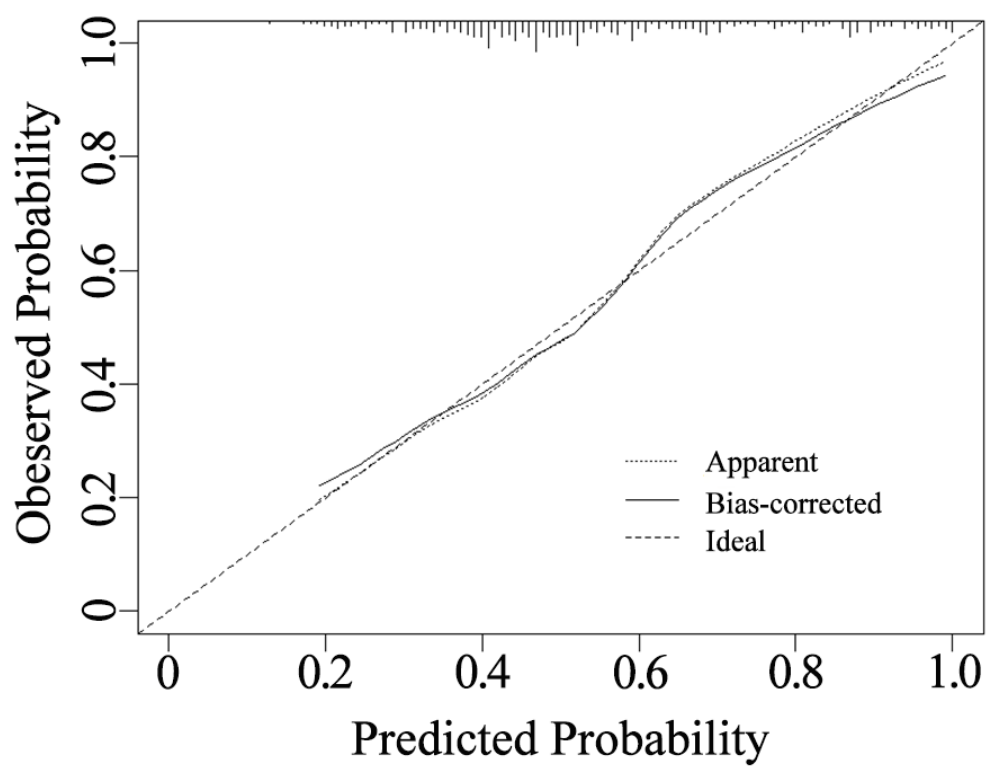

B = 1000 repetitions, boot

Mean absolute error = 0.019 n=297

Supplement: Supplemental Information 2 [file peerj-12-18454-s002.zip › Figures and data/Figure4/Fgures4.pdf]

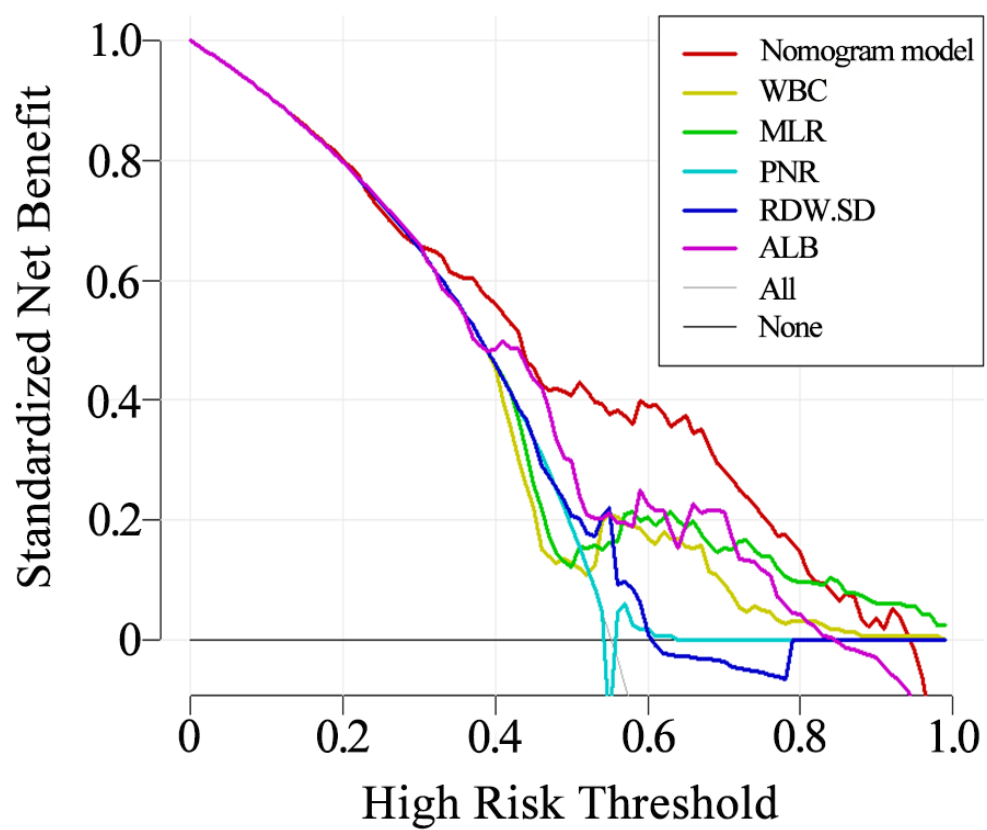

Supplement: Supplemental Information 2 [file peerj-12-18454-s002.zip › Figures and data/Figure5/Fgure5.pdf]
